# An echocardiographic prognostic risk stratification decision tree to determine adverse events in Anderson-Fabry disease

**DOI:** 10.1093/ehjimp/qyaf032

**Published:** 2025-05-08

**Authors:** Luke Stefani, Anita Boyd, Jennifer Pham, Matthew Zada, Peter Emerson, Kerry Devine, Michel Tchan, Liza Thomas

**Affiliations:** Westmead Clinical School, University of Sydney, Westmead Hospital, Westmead, 2145 NSW, Australia; Department of Cardiology, Westmead Hospital, Westmead, 2145 NSW, Australia; Westmead Private Cardiology, Westmead, 2145 NSW, Australia; Department of Cardiology, Westmead Hospital, Westmead, 2145 NSW, Australia; Westmead Clinical School, University of Sydney, Westmead Hospital, Westmead, 2145 NSW, Australia; Department of Cardiology, Westmead Hospital, Westmead, 2145 NSW, Australia; Westmead Clinical School, University of Sydney, Westmead Hospital, Westmead, 2145 NSW, Australia; Department of Cardiology, Westmead Hospital, Westmead, 2145 NSW, Australia; Department of Genetic Medicine, Westmead Hospital, Westmead, 2145 NSW, Australia; Westmead Clinical School, University of Sydney, Westmead Hospital, Westmead, 2145 NSW, Australia; Department of Genetic Medicine, Westmead Hospital, Westmead, 2145 NSW, Australia; Westmead Clinical School, University of Sydney, Westmead Hospital, Westmead, 2145 NSW, Australia; Department of Cardiology, Westmead Hospital, Westmead, 2145 NSW, Australia; Southwestern Clinical School, University of New South Wales, Sydney, 2170 NSW, Australia

**Keywords:** Fabry, speckle tracking, echocardiography, adverse events, risk decision tree

## Abstract

**Aims:**

Anderson–Fabry disease (AFD) is an X-linked disease, with cardiac involvement resulting in increased left ventricular (LV) wall thickness. Speckle tracking echocardiography analysis may be more sensitive in the assessment of myocardial impairment in AFD patients and have prognostic value. Our aim was to evaluate LV and left atrial (LA) dysfunction by traditional and strain parameters in AFD patients and evaluate prognostic utility.

**Methods and results:**

Fifty-six AFD patients were age- and sex-matched to 56 healthy controls. LV global longitudinal strain (GLS) and LA reservoir strain (LAS_R_) were significantly lower in male (GLS: 19.38[3.21] vs. 17.8[7.0], *P* = 0.009; LAS_R_: 38.07 ± 6.67 vs. 31.12 ± 6.76, *P* = 0.003) and female (GLS: 20.58 ± 1.63 vs. 19.29 ± 1.67, *P* = 0.003; LAS_R_: 38.77 ± 7.43 vs. 33.13 ± 6.06, *P* < 0.001) AFD patients compared with controls. Reduced strain parameters were also seen in female AFD patients with normal wall thickness (GLS: 20.88 ± 1.74 vs. 19.72 ± 1.53, *P* = 0.037; LAS_R_: 40.09 ± 7.15 vs. 34.79 ± 6.20, *P* = 0.004). 53/56 AFD patients had a median follow-up of 43[81] months; 11/53 experienced an adverse cardiovascular event (i.e. cardiac death, myocardial infarction, arrhythmias, stroke. and heart failure). LV wall thickness, LAVI_max_, and LV GLS displayed good sensitivity and specificity for adverse cardiac events. A prognostic risk decision tree comprising of these parameters demonstrated good predictive value for adverse events (AUC = 0.910).

**Conclusion:**

We demonstrate differences in LV and LA echocardiographic parameters in AFD patients compared with healthy controls, including female AFD patients with normal LV wall thickness. A prognostic risk decision tree stratified AFD patients into three groups with the highest risk group demonstrating more AFD-related adverse events.

## Graphical Abstract

**Graphical Abstract:**
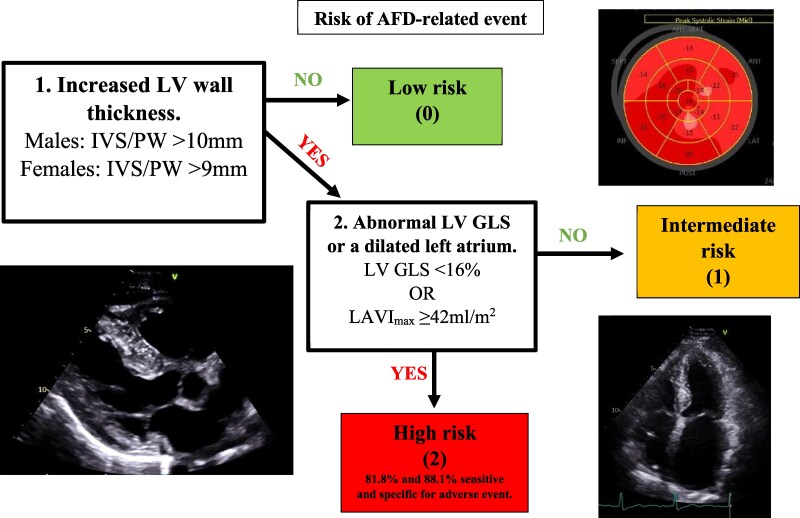
An echocardiographic prognostic risk decision tree to determine adverse events was developed using 56 AFD patients from a single centre. Those that had normal wall thickness were classed as low risk (0). If a patient had increased wall thickness, they were classed as intermediate risk (1). If a patient had increased wall thickness and abnormal LV GLS or moderately/severely dilated left atrium they were classed as high risk (2). Patients classed as low and intermediate risk had better outcomes, with 0/28 and 2/11 (18.2%) experiencing an AFD-related event respectively. Patients classed as high risk performed worse with 9/14 (64.3%) experiencing an AFD-related event.

## Introduction

Anderson-Fabry disease (AFD) is an X-linked, lysosomal storage disease caused by α-galactosidase A enzyme deficiency,^1^ that results in the accumulation of sphingolipids in multiple organs. The resulting clinical manifestations of AFD range from renal, gastrointestinal, neurologic, and cardiac dysfunction. The heart is one of the most frequently involved organs, with structural changes including increased left ventricular (LV) wall thickness, with consequent fibrosis and ultimately heart failure and arrhythmias.^2,3^ Given AFD is X-linked, females were historically considered only disease carriers; however, it is now widely accepted that women can also develop AFD-related cardiomyopathy.^4^ Additionally, increased wall thickness of AFD patients has been linked to overall cardiac morbidity and mortality.^5^ The increased use of 2-dimensional speckle tracking echocardiography (2D-STE) derived LV global longitudinal strain (GLS) and left atrial (LA) strain, may be advantageous in the assessment of early myocardial impairment in AFD patients. The utilization of these 2D-STE parameters in addition to traditional parameters could provide clinicians greater insight into AFD disease stage, determine progression and enable treatment delegation. In this cross-sectional study, we evaluated traditional and 2D-STE strain parameters in patients with AFD and compared them with age- and sex-matched healthy controls. Additionally, AFD patients were followed up for adverse cardiovascular events to examine the prognostic utility of echocardiographic parameters.

## Methods

A total of 64 AFD patients were retrospectively identified from our hospital database, which is the state referral centre, with AFD patients having regular cardiac follow-up at our tertiary hospital. The study protocol was approved by the local ethics committee (WSLHD HREC no. 2019/ETH13628). All individuals underwent clinical examination and a comprehensive transthoracic echocardiogram (TTE). Eight patients were excluded from analysis due to poor image quality or due to patients being on enzyme replacement treatment (ERT) for >2 years prior to their index echocardiogram. It was felt that a period of ERT exceeding 2 years prior to the index TTE may alter the cardiac manifestations. The remaining 56 AFD patients [ERT naïve (*n* = 49) or within 2 years of commencing therapy (*n* = 7)] were age- (±5 years) and sex-matched with 56 healthy controls from a departmental database. Healthy controls were defined as individuals with no cardiovascular risk factors or history of ischaemic, structural or valvular heart disease. AFD-related adverse events included a composite of cardiac death, myocardial infarction, arrhythmias, cerebral vascular accident/transient ischaemic attacks, and admission for heart failure. The outcome data were obtained through the review of patient medical records, with follow-up taken as time from the index echocardiogram.

A comprehensive TTE was performed using commercially available ultrasound machines (Vivid E90, General Electric Healthcare, Horton, Norway). All TTEs were performed by experienced medical professionals or cardiac sonographers. Images were obtained with subjects in left-lateral decubitus position, and acquired from parasternal, apical, and sub-costal views using a 3.5-MHz transducer and acquired at high frame rates (>55 fps).^6^ All patients were in sinus rhythm at the time of scan. Measurements and recordings were obtained according to international recommended guidelines.^7–11^ Analysis was performed offline using dedicated software (EchoPac v 203, General Electric-Vingmed), with the investigator blinded to age and sex of the subject.

Traditional LV measurements were obtained including biplane LV end-diastolic and end-systolic volumes and indexed to body surface area.^7^ Biplane LV ejection fraction was calculated according to Simpson’s method. LV mass was calculated using the Devereaux method and indexed to body surface area.^9^ Increased wall thickness was classified based on ASE guidelines as interventricular septum (IVS) and posterior wall (PW) being >10 mm for males and >9 mm for females, in diastole.^7^ Average LV wall thickness was calculated as the average thickness between IVS and PW, in diastole. Trans-mitral pulsed-wave Doppler from the apical 4-chamber view was used to obtain mitral inflow velocities to assess LV diastolic filling, with the sample volume placed at the mitral leaflet tips.^10^ Measurements of mitral inflow included the peak velocities of early (peak E) and late diastolic filling (peak A), and the E/A ratio. Pulsed tissue Doppler imaging was performed placing the sample volume at the septal and lateral mitral annulus in the apical 4-chamber view,^10,11^ obtaining early diastolic (e′) annular velocity. An average of septal and lateral annular e′ velocity was obtained, as recommended.^11^ The E/e′ was calculated using average e′.^10^ LA volume was calculated by modified Simpson’s biplane method of discs from zoomed LA focused apical 4- and 2-chamber views. LA maximal volume (LAVI_max_) was measured at the end of LV systole and LA minimum volume (LAVI_min_) measured at the end of LV diastole, with both indexed to body surface area.^7^ LA function was assessed by LA ejection fraction (LAEF): ((LAVI_max_ − LAVI_min_)/LAVI_max_) *100.

**Figure 1:**
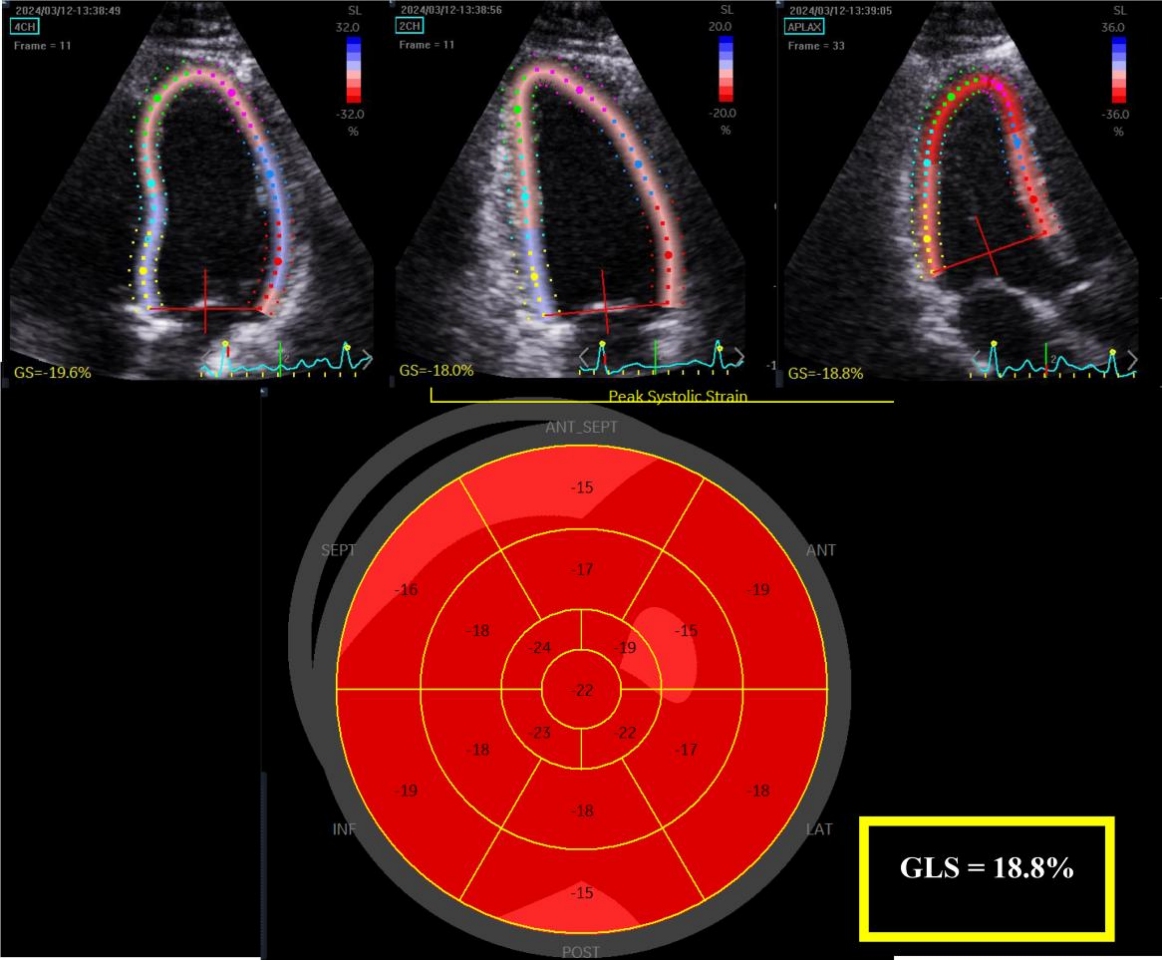
The endocardial border of the left ventricle was traced at end-systole, with strain calculated as the average of the peak negative global strain during systole from the 4-, 2-, and 3-chamber views.

**Figure 2:**
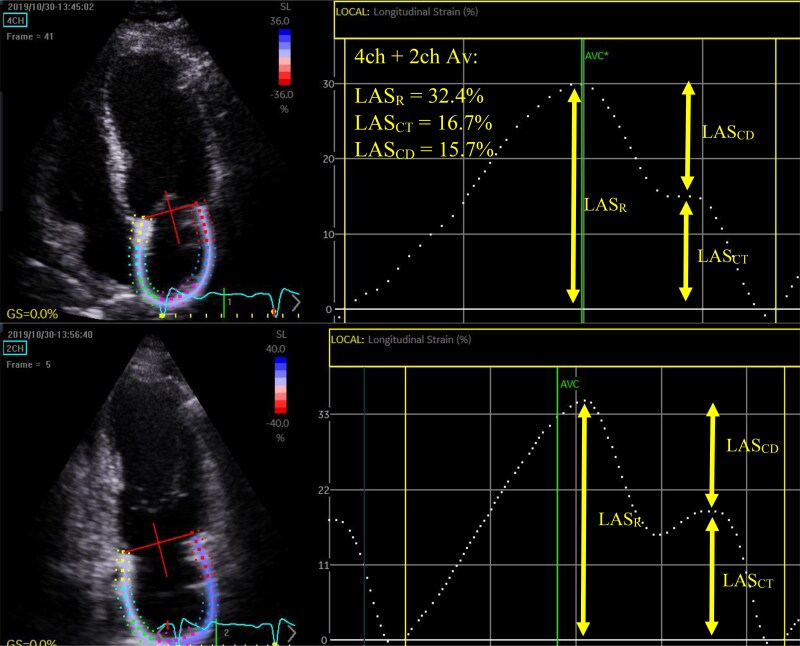
Speckle tracking strain analysis of left atrium. LAS_R_ was measured as the peak positive systolic strain, and contractile strain LAS_CT_ as the peak strain just before late diastolic contraction of the LA. Conduit strain LAS_CD_ was calculated by subtracting LAS_CT_ from LAS_R_.

LV and LA strain measurements were measured offline using 2D-STE software allowing semiautomated analysis (Q analysis, EchoPac v 203, General Electric Healthcare, Horton, Norway). All strain measurements were performed using R-R wave gating. LV GLS was measured according to published guidelines.^7^ LV GLS was measured offline from the 3 apical LV focused views acquired at high frame rates (>55 fps). The endocardial border of the left ventricle was traced in end-systole and the region of interest was set to include the LV myocardium. LV systolic strain was measured as the average of the peak negative global strain during systole from the 4-, 2-, and 3-chamber views (*Figure 1*). Although LV GLS produces ‘negative’ values to represent myocardial shortening, for simplicity, the absolute values of GLS are reported in the results. 2D-STE LA phasic strain analysis has been previously described^12,13^ (*Figure 2*). LA reservoir strain (LAS_R_) was measured as the peak positive systolic strain, and contractile strain (LAS_CT_) as the peak strain just before late diastolic contraction of the LA. Conduit strain (LAS_CD_) was calculated by subtracting LAS_CT_ from LAS_R_. Of the 56 patients included in the final analysis, 4 could not have LA strain measured due to poor image quality.

Analyses were performed using IBM SPSS Statistics version 26 (SPSS, Chicago, IL, USA). Continuous variables are summarized using mean ± standard deviation and median [interquartile range (IQR)], depending on data distribution, and categorical variables using number and percentages. Kolmogorov–Smirnov test was used to determine if samples were normally distributed. Paired Student’s *t*-test and nonparametric Wilcoxon signed-ranks test were used where appropriate for continuous variable comparison. Pearson χ^2^ test was used for categorical variable comparison. Outcome data were obtained through patient medical records with follow-up duration taken as the time from TTE to the first AFD-related adverse cardiovascular event or till last date of follow-up. Receiver-operator characteristic (ROC) curves were used to determine sensitivity and specificity of parameters to predict AFD-related events. Paired-sample design test was used to compare ROC curves. Kaplan–Meier plots were used to illustrate AFD-related event function within the cohort. Values were considered significant if *P* < 0.05.

Inter-observer variability for LV GLS and LA strain parameters was performed by 2 independent operators blinded to measurements in 10 randomly selected patients. Intra-observer variability was performed by the same operator in the same 10 patients at least 4 weeks after the initial measurements. Variability was evaluated by intraclass correlation coefficients (ICC) using a 2-way random effects model and a 95% confidence interval; values between 0.75 and 0.9 represented good reliability and values ≥0.90 excellent reliability.^14^

## Results

Fifty-six patients with AFD and 56 age- and sex-matched controls were included in the final analysis. Demographic data are outlined in *Table 1* and Supplementary data online, *Table S1*. Systolic blood pressure was significantly higher in the controls, although within normal range. All other demographic data were similar between the 2 groups. As expected, female AFD patients had fewer cardiovascular risk factors and major adverse cardiovascular events compared with males (see Supplementary data online, *Table S1*). Echocardiographic parameters of controls and AFD groups are outlined in *Table 2* (males) and *Table 3* (females).

**Table 1:** Demographic data of control and AFD groups at time of TTE

	Controls *n* = 56	AFD *n* = 56	*P* value
Age (years)	41.1 ± 14.4	40.9 ± 14.1	0.572
Height (cm)	168.0 ± 9.9	168.6 ± 8.8	0.656
Weight (kg)	71.8 [24.5]	70.0 [18.6]	0.497
BMI (kg/m^2^)	24.7 [7.1]	23.5 [7.0]	0.443
BSA (m^2^)	1.77 [0.23]	1.77 [0.24]	0.842
SBP (mmHg)	124 [12]	120 [22]	0.003
DBP (mmHg)	78 [7.8]	74 [7.1]	0.019
Hypercholesterolemia/Hyperlipidaemia	7 (12.5)	17 (30.3)	0.021
Smoker	12 (21.4)	11 (19.6)	0.815
Diabetes	0	4 (7.1)	N/A
Hypertension	0	13 (23.2)	N/A
Chronic kidney disease	0	9 (16.1)	N/A
Angina	0	10 (17.9)	N/A
Myocardial infarction	0	2 (3.6)	N/A
Heart failure	0	4 (7.1)	N/A
Ventricular tachycardia	0	1 (1.8)	N/A
Atrial fibrillation	0	2 (3.6)	N/A
CVA or TIA	0	3 (5.4)	N/A
eGFR	N/A	78.45 ± 21.24	N/A
ERT treatment at time of scan	N/A	7 (12.5)	N/A

Mean ± standard deviation or median [IQR] for continuous variables; number (percentage) for categorical variables.

BMI, body mass index; BSA, body surface area; CVA, cardiovascular accident; DBP, diastolic blood pressure; SBP, systolic blood pressure; TIA, transient ischaemic attack.

**Table 2:** Comparison of echocardiographic parameters between male control and male AFD patients

	Controls *n* = 22	AFD *n* = 22	*P* value
Age (years)	42.1 ± 13.4	42.9 ± 13.9	0.125
IVS (mm)	9.0 [1.8]	12.9 [5.6]	<0.001
PW (mm)	9.0 [1.0]	11.1 [3.8]	0.001
LVMI (g/m^2^)	71.4 [26.5]	122.4 [52.8]	<0.001
LVEDVI (mL/m^2^)	48.7 [12.0]	63.1 [34.6]	<0.001
LVESVI (mL/m^2^)	19.0 [7.7]	26.8 [15.2]	0.001
LVEF (%)	61.4 ± 5.3	57.3 ± 9.1	0.061
E/A	1.4 ± 0.3	1.7 ± 0.8	0.042
Septal e′ (cm/s)	10.3 ± 2.0	7.6 ± 2.8	<0.001
Lateral e′ (cm/s)	13.2 ± 2.6	10.4 ± 3.8	0.005
Average E/e'	6.0 [2.9]	8.7 [2.4]	<0.001
LAVI_min_ (mL/m^2^)	11.8 ± 2.7	18.5 ± 5.6	<0.001
LAVI_max_ (mL/m^2^)	26.1 ± 4.9	43.0 ± 12.2	<0.001
LAEF (%)	54.6 ± 7.9	56.0 ± 7.2	0.352
LV GLS (%)	19.38 [3.21]	17.8 [7.0]	0.009
LAS_R_ (%)	38.07 ± 6.67	31.12 ± 6.76	0.003
LAS_CT_ (%)	17.22 ± 3.02	13.15 ± 4.13	0.001
LAS_CD_ (%)	20.86 ± 5.81	17.98 ± 6.38	0.102

Mean ± standard deviation or median [IQR] for continuous variables.

GLS, global longitudinal strain; IVS, interventricular septum; LAEF, left atrial ejection fraction; LAS_CD_, left atrial conduit strain; LAS_CT_, left atrial contractile strain; LAS_R_, left atrial reservoir strain; LAVI_max_, maximum left atrial indexed volume; LAVI_min_, minimum left atrial indexed volume; LV, left ventricular; LVEDVI, left ventricular end-diastolic volume indexed; LVEF, left ventricular ejection fraction; LVESVI, left ventricular end-systolic volume indexed; LVMI, left ventricular mass indexed; PW, posterior wall.

**Table 3:** Comparison of echocardiographic parameters between female control and all female AFD and female AFD sub-group with normal LV wall thickness

	Entire female AFD cohort			Female AFD cohort with normal LV wall thickness		
	Controls *n* = 34	AFD *n* = 34	*P* value	Controls *n* = 23	AFD *n* = 23	*P* value
Age (years)	40.44 ± 15.24	40.62 ± 14.24	0.709	37.17 ± 14.93	37.61 ± 14.36	0.444
IVS (mm)	7.00 [2.6]	7.88 [3.25]	0.020	7.00 [2.60]	7.00 [1.27]	0.851
PW (mm)	7.63 [1.43]	8 [3.46]	0.032	7.40 [1.00]	7.00 [2.00]	0.658
LVMI (g/m^2^)	62.22 [17.56]	63.36 [35.83]	0.052	60.53 ± 13.29	58.75 ± 13.37	0.690
LVEDVI (mL/m^2^)	43.78 ± 10.45	54.59 ± 12.94	<0.001	43.94 ± 10.76	54.71 ± 12.49	0.004
LVESVI (mL/m^2^)	16.66 ± 3.97	21.11 ± 6.71	0.001	16.87 ± 3.79	21.17 ± 6.35	0.010
LVEF (%)	61.82 ± 4.91	61.78 ± 5.75	0.972	61.17 ± 5.04	61.67 ± 6.08	0.752
E/A	1.39 [0.64]	1.50 [0.58]	0.264	1.37 [0.74]	1.63 [0.90]	0.108
Septal e′ (cm/s)	9.48 ± 2.59	9.70 ± 3.06	0.662	10.00 ± 2.59	10.62 ± 2.76	0.286
Lateral e′ (cm/s)	12.44 ± 3.35	12.32 ± 4.22	0.847	12.95 ± 3.70	13.82 ± 4.04	0.287
Average E/e'	7.36 [2.61]	7.32 [4.21]	0.654	7.08 [2.48]	7.03 [2.26]	0.741
LAVI_min_ (mL/m^2^)	11.25 [2.47]	14.72 [8.92]	0.002	10.81 ± 2.90	13.85 ± 6.16	0.042
LAVI_max_ (mL/m^2^)	26.76 ± 6.22	32.37 ± 10.12	0.007	26.30 ± 6.45	30.90 ± 10.25	0.072
LAEF (%)	58.39 ± 7.15	53.06 ± 11.26	0.012	58.80 ± 6.69	55.33 ± 11.61	0.133
LV GLS (%)	20.58 ± 1.63	19.29 ± 1.67	0.003	20.88 ± 1.74	19.72 ± 1.53	0.037
LAS_R_ (%)	38.77 ± 7.43	33.13 ± 6.06	<0.001	40.09 ± 7.15	34.79 ± 6.20	0.004
LAS_CT_ (%)	15.08 ± 3.91	12.92 ± 2.64	0.017	15.26 ± 3.87	12.20 ± 2.52	0.009
LAS_CD_ (%)	23.69 ± 8.67	20.21 ± 6.18	0.028	24.83 ± 8.88	22.59 ± 5.72	0.220

Mean ± standard deviation or median [IQR] for continuous variables.

GLS, global longitudinal strain; IVS, interventricular septum; LAEF, left atrial ejection fraction; LAS_CD_, left atrial conduit strain; LAS_CT_, left atrial contractile strain; LAS_R_, left atrial reservoir strain; LAVI_max_, maximum left atrial indexed volume; LAVI_min_, minimum left atrial indexed volume; LV, left ventricular; LVEDVI, left ventricular end-diastolic volume indexed; LVEF, left ventricular ejection fraction; LVESVI, left ventricular end-systolic volume indexed; LVMI, left ventricular mass indexed; PW, posterior wall.

LV wall thickness and volumes were significantly higher in AFD patients when compared with controls. This was evident in both male and female patients. Whilst the mean LV end-diastolic indexed volume (LVEDVI) was within normal range, 9 (26.5%) of the female AFD patients, and 8 (36.4%) of the male AFD patients had an enlarged LVEDVI, as per ASE guidelines. LVEF was similar in both male and female patients. Doppler-derived diastolic parameters were not significantly different between female controls and female AFD patients, whereas male AFD patients demonstrated lower septal and lateral e′ values with a subsequent higher average E/e′ when compared with male controls. LA volumes were significantly larger in both male and female AFD groups when compared with controls. LV GLS was significantly lower in male and female AFD patients compared with controls, albeit with mean GLS within normal range for the female cohort. LAS_R_ and LAS_CT_ were also significantly lower in the male and female AFD group compared with controls, whilst LAS_CD_ was similar. The sub-group of female AFD patients with normal wall thickness was compared with controls (*Table 3*). LVEDVI, LV end-systolic indexed volume (LVESVI), and LAVI_min_ were significantly larger even in this subset of female AFD patients, whereas LAVI_max_ was not. All LV and LA 2D-STE strain parameters remained significantly lower when compared with controls.

**Figure 3:**
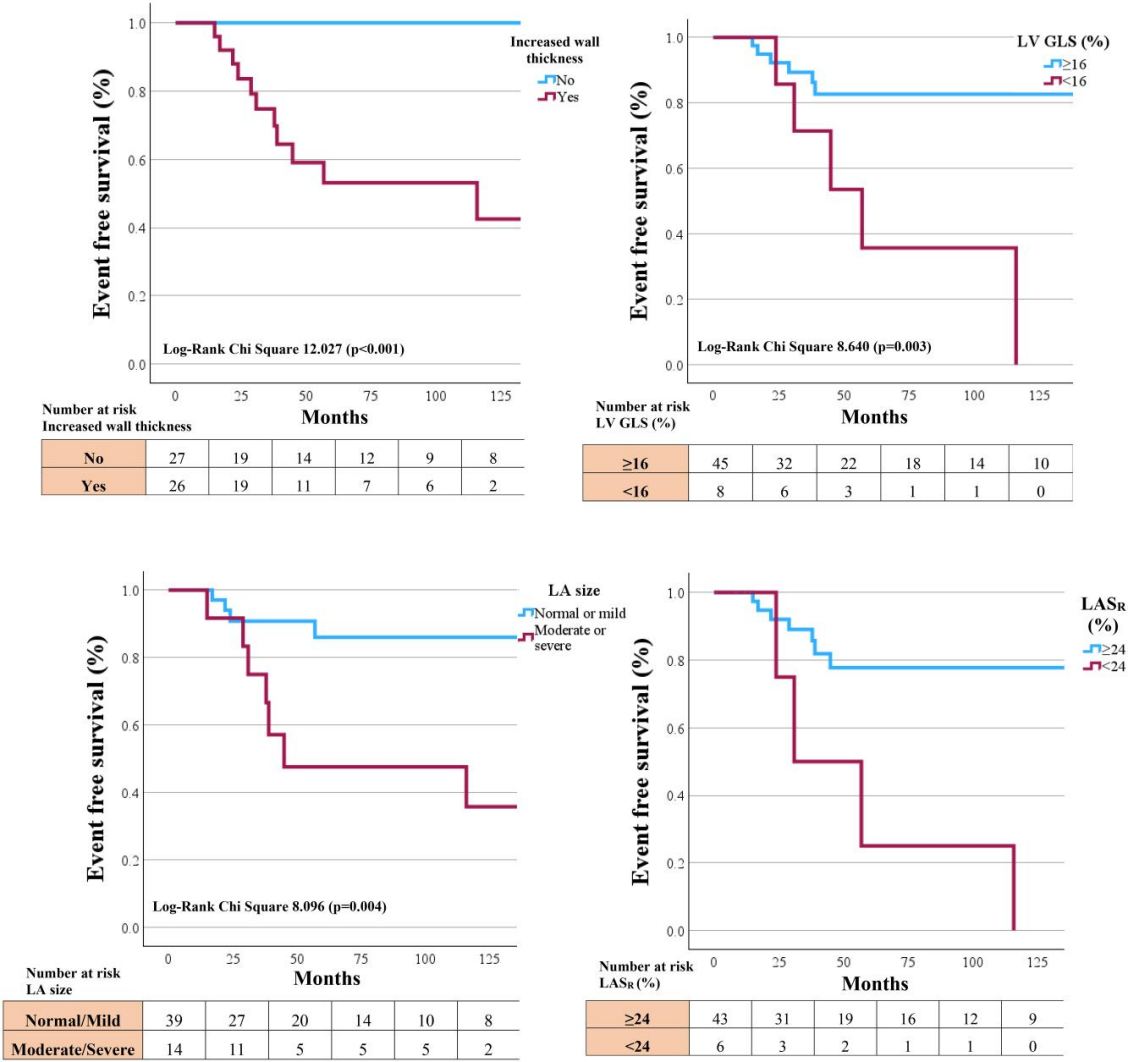
Kaplan–Meier curves for (*A*) LVEDVI, (*B*) LAVI_max_, (*C*) LV GLS, and (*D*) LAS_R_.

During a median follow-up of 43[81] months, 53/56 AFD patients had follow-up data with 11/53 having a reported AFD-related adverse cardiovascular event. ROC analysis was performed on echocardiographic parameters to determine suitability in predicting AFD-related adverse events (*Table 4*). Average LV wall thickness (AUC = 0.90), LAVI_max_ (AUC = 0.83), LV GLS (AUC = 0.81) and LAS_R_ (AUC = 0.79) demonstrated good sensitivity and specificity. We evaluated these parameters as binary variables, using clinically utilized cut-off values for each parameter to evaluate their impact on AFD-related end points. Increased wall thickness (>10 mm in males and >9 mm in females) as per ASE guidelines,^7^ LAVI_max_ if greater than moderately dilated (>42 mL/m^2^) as per ASE guidelines,^7^ LV GLS was defined as abnormal if <16%,^15^ and LAS_R_ as abnormal if <24%, corresponding to previously reported values.^16,17^ Stratification by clinical ‘normal’ values for the 4 parameters demonstrated increased wall thickness (*P* < 0.001), LV GLS (*P* = 0.003), LAVI_max_ (*P* = 0.004), and LAS_R_ (*P* = 0.002) as significant predictors of AFD-related adverse events on Kaplan–Meier analysis (*Figure 3*). Of the 11 AFD patients that had an AFD-related event, all had some degree of increased wall thickness, 5/11 (45.4%) had abnormal LV GLS, 7/11 (63.6%) had at least moderately dilated left atrium and 4/11 (36.4%) had an abnormal LA strain value.

**Table 4:** Receiver operating curve analysis of univariate predictors of AFD-related events

Parameter	AUC	*P* value
Av LV wall thickness	0.90	<0.001
LAVI_max_	0.83	<0.001
LVEDVI	0.69	0.041
E/e′	0.73	0.024
LVEF	0.65	0.038
LAS_R_	0.79	<0.001
LV GLS	0.81	<0.001

AUC, area under curve; Av, average; GLS, global longitudinal strain; LAS_R_, left atrial reservoir strain; LAVI_max_, maximum left atrial indexed volume; LV, left ventricular; LVEDVI, left ventricular end-diastolic volume indexed; LVEF, left ventricular ejection fraction.

**Figure 4:**
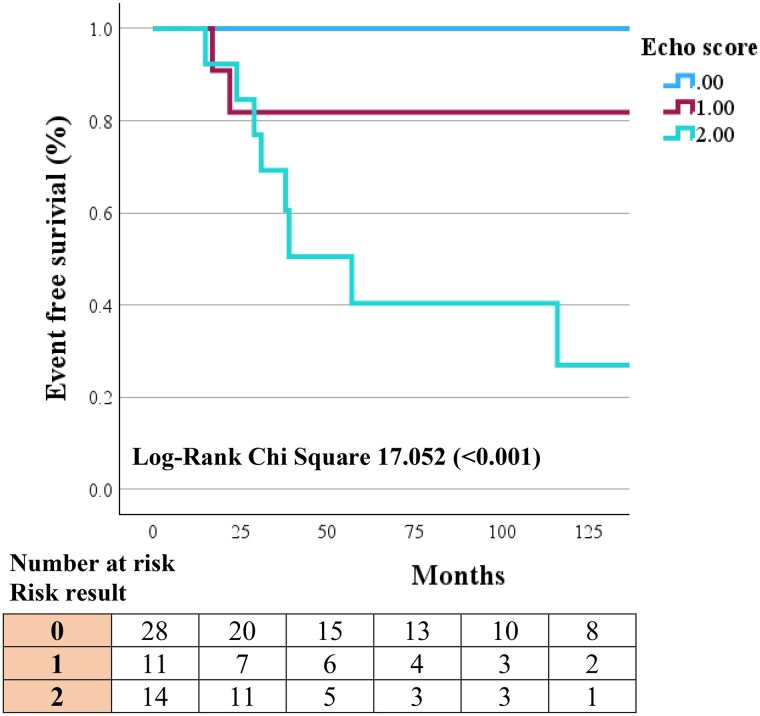
Kaplan–Meier curve for novel echocardiographic risk decision tree. Patients with increased wall thickness and abnormal LV GLS or moderately/severely dilated left atrium were classed as high risk (2). Patients with only increased wall thickness, were classed as intermediate risk (1). Patients with normal wall thickness were classed as low risk (0).

Using these echocardiographic parameters, a simple prognostic risk stratification decision tree was constructed to predict those at highest risk of experiencing an AFD-related event (see *Graphical Abstract*). LAS_R_ was not included in the final analysis due to collinearity with LAVI_max_. Since increased wall thickness encompassed all AFD-related events, those that did not have increased wall thickness were classed as low risk (0). If a patient had increased wall thickness, they were classed as intermediate risk (1). If a patient had increased wall thickness, and either abnormal LV GLS or moderately/severely dilated left atrium, they were classed as high risk (2). Kaplan–Meier curves were derived using this novel echocardiographic risk stratification decision tree (*Figure 4*). Patients classed as low and intermediate risk had better outcomes, with 0/28 and 2/11 (18.2%) experiencing an AFD-related event, respectively. Patients classed as high risk performed worse with 9/14 (64.3%) experiencing an AFD-related adverse event. This risk stratification decision tree demonstrated good discriminatory value with an AUC of 0.91. The sensitivity and specificity for a patient classed as high risk and having an adverse event was 81.8% and 88.1%, respectively.

For intra-observer variability, the ICC for LAS_R_ was 0.96 (0.83–0.99), LAS_CT_ was 0.97 (0.86–0.99), LAS_CD_ was 0.97 (0.88–0.99), LV GLS was 0.94 (0.78–0.99), LAVI_max_ was 0.99 (0.96–0.99), IVS was 0.99 (0.98–0.99), and PW was 0.98 (0.91–0.99). The inter-observer variability for LAS_R_ was 0.97 (0.88–0.99), LAS_CT_ was 0.98 (0.92–0.99), LAS_CD_ was 0.98 (0.91–0.99), LV GLS was 0.96 (0.84–0.99), LAVI_max_ was 0.94 (0.76–0.99), IVS was 0.97 (0.89–0.99), and PW was 0.97 (0.86–0.99). The results here demonstrate good reproducibility for all parameters.

## Discussion

In this retrospective cross-sectional study, we compared 56 AFD patients with age- and sex-matched healthy controls and evaluated clinical, traditional echocardiographic parameters, LV, and LA strain parameters. The key findings from this study include:

1. LV structural (LV volumes) and functional (LV GLS) changes occur in both female and male AFD patients, despite normal LV wall thickness.
2. LV wall thickness, LV GLS, LAVImax, and LASR, stratified by clinical cut-offs, demonstrated worse outcomes with worsening values.
3. A composite echocardiographic risk stratification decision tree comprising of LV wall thickness, LV GLS, and LAVImax demonstrated good predictive value for AFD-related adverse events.

Increased LV wall thickness is the predominant cardiovascular manifestation seen in AFD patients, however various other structural (increased RV free wall thickness, LA or bi-atrial dilation, valve leaflet thickening, aortic root, and ascending aorta dilation) and functional (systolic and diastolic dysfunction, GLS impairment) features have been identified.^18–21^ These manifestations have also been reported in female AFD patients also.^22,23^ Whilst these features were evident in this AFD cohort, we also report higher LV volumes in both male and female AFD patients when compared with healthy age- and sex-matched controls, despite AFD patients not having significantly reduced LVEF. Interestingly, this difference in LV volumes is also true for AFD female patients without increased wall thickness when compared with controls. This suggests that structural changes (LV volumes) may occur in AFD patients prior to the development of clinically overt functional changes (LVEF). LV dilation in AFD patients has been previously reported in studies using cardiac magnetic resonance.^24,25^

Echocardiographic parameters have previously attracted interest as prognostic tools in predicting AFD-related adverse events, with many studies primarily focusing on LV structure and function. A recent study by Chang et al. highlights the significance of increased LV wall thickness and LV GLS as prognostic tools in AFD patients.^26^ They demonstrated that grades of LV wall thickness predict MACE rates, with severe increase in wall thickness exhibiting a 50.5% MACE rate.^26^ Further investigation of LV sub-clinical function by use of LV GLS demonstrated that those with severely increased wall thickness and impaired LV GLS had the highest incidence of MACE, irrespective of sex, genotype and irrespective of whether the patient was receiving ERT.^26^ Our group previously demonstrated that impaired LV GLS, even in those with normal LV wall thickness, had a strong association with AFD-related adverse events.^21^ Additionally, previous reports have suggested that LA functional and structural impairment may precede LV functional and structural changes.^27–29^ This was suggested in our AFD cohort, with all patients having abnormal LV GLS also having a reduced LAS_R_ <24% or increased LAVI_max_.^7,16,17^ Despite this there are scarce reports of LA parameters as predictors of adverse events in AFD patients. Pichette et al. assessed LA volumetric and strain parameters in 51 AFD patients prior to ERT. At a median follow-up period of 50 months, 5 patients developed new-onset AF and 4 patients had a stroke. On univariate analysis, they demonstrated LAS_R_ and LV GLS as the most significant factors associated with these events.^30^ The results from these studies demonstrate that both LV and LA parameters have strong prognostic utility, and that the early introduction of treatment can result in reversal of atrial myopathy.

In our study, we demonstrate that by combining LV structural and functional and LA structural parameters, we can construct a composite risk stratification decision tree, which is more accurate in identifying patients at risk of experiencing an AFD-related adverse event. All 11 patients with an AFD-related adverse event had some degree of increased LV wall thickness, highlighting the significant sensitivity that increased LV wall thickness has in the prediction of AFD-related adverse events (AUC = 0.821; Supplementary data online, *Table S2*). This however lacked specificity given 15/26 (57.7%) patients with increased wall thickness did not have an AFD-related adverse event. To improve this, an additional level was added to the decision tree whereby those with an abnormal LV GLS or a dilated LA were classed as high risk. This improved performance with the constructed echocardiographic risk decision tree demonstrating better prognostic valve than just increased LV wall thickness alone (AUC 0.910 vs. AUC 0.821; *P* = 0.013). LAS_R_ was considered in initial analysis as an additional substrate in the prediction of outcomes however was excluded due to collinearity and due to the superiority of LAVI_max_ in this cohort (abnormal LAVI_max_ in 7/11 AFD patients with adverse event; abnormal LAS_R_ in 4/11 AFD patients with adverse event). Moreover, LAVI_max_ has been previously utilized for staging AFD as discussed below.^31^

Staging of AFD progress by echocardiographic parameters has been previously reported by Meucci et al.^31^ This group categorises AFD patients into 4 stages: stage 0, no cardiac involvement; stage 1, LV hypertrophy (LV maximal wall thickness >12 mm); stage 2, left atrium (LA) enlargement (LA volume index >34 mL/m^2^); stage 3, ventricular impairment (LV ejection fraction <50% or E/eʹ ≥ 15 or TAPSE <17 mm). As expected, patients in higher stages have worse structural and functional echocardiographic parameters and reported a greater risk of presenting with an adverse cardiac event.^31^ Only traditional echocardiographic measurements are utilized in the staging process which can be advantageous as they are well established. However, given the results presented in our study, the addition of LV GLS may prove beneficial particularly for those at risk of progressing from stage 2 into stage 3. The sensitivity of LV GLS to detect early sub-clinical LV dysfunction is well known, with a reduction of LV GLS suggesting possible future LV functional impairment (reduced LVEF).^15,32,33^ Thus, it may be valuable to include LV GLS so physicians are able to identify AFD patients at risk and provide earlier intervention.

Given the systemic effects AFD has on both the left ventricle and left atrium, the creation of an echocardiographic risk stratification decision tree using parameters of both LV and LA parameters demonstrated improved prognostic utility in the prediction of AFD-related adverse events. The use of an echocardiographic risk stratification decision tree, such as this may also assist clinicians in the early commencement of ERT to prevent adverse events. Whilst relatively simple, validation of this novel echocardiographic risk stratification decision tree is required in a different cohort with longitudinal follow-up for adverse events, before its use can be recommended for monitoring and patient risk stratification.

### Clinical relevance

Therapeutic intervention early in the course of AFD may slow or even result in reverse remodelling, as presented by Pichette et al.^30^ In many countries, including Australia, ERT is only commenced when there is overt LV structural change. Thus, early identification of LV dysfunction and LA enlargement is likely to have clinical importance in terms of being an additional marker for commencement of ERT. This study additionally demonstrates that the use of both LA and LV parameters for prognostic outcome is effective and may be potentially useful to clinicians in patient management.

### Limitations

The number of AFD patients and AFD-related events (*n* = 11) included was modest; however, given the prevalence of AFD, the sample size is reasonable, particularly from a single centre. Despite this we demonstrate an association between abnormal LA parameters and AFD-related adverse events. Additionally, because of the small number of adverse events, we were unable to perform comparisons of multiple echocardiographic parameters to predict events. Given the clinical course of AFD, majority of events occurred in male patients; however, it would be interesting to assess predictors of AFD-related adverse events specifically in a female AFD cohort. Further validation in another Fabry cohort would greatly increase the echocardiographic risk stratification decision tree’s reliability.

Two patients with a previous myocardial infarct were included in the final analysis, the implication being that impaired LV GLS may be as a result of infarct rather than AFD cardiomyopathy. These 2 patients were included in final analysis as we performed this as a ‘real world’ study and due to the limited number of events within the AFD cohort. Future analysis should be considered within a larger AFD cohort where the ability to exclude these patients is feasible. We did not include RV parameters given the small sample size included in this study.

## Conclusion

We demonstrated changes in LA size, LA, and LV strain despite preserved LV function (i.e. normal LVEF); importantly these ‘sub-clinical’ cardiac functional changes were also noted in female AFD patients with normal LV wall thickness. Use of a composite echocardiographic risk stratification decision tree stratified AFD patients into three groups with the highest risk demonstrating improved predictive value for AFD-related adverse events. Future longitudinal validation studies with larger number of AFD patients are required to determine its utility.

## Supplementary Material

qyaf032_Supplementary_Data

## Data Availability

The data underlying this article will be shared on reasonable request to the corresponding author.

## Lead Author Biography

Luke Stefani is a cardiac sonographer at Westmead Hospital in Sydney, Australia, and a PhD candidate at the University of Sydney. His research interests include speckle tracking echocardiography, infiltrative cardiomyopathies, and diastolic dysfunction.
