# Supplementary material for: An echocardiographic prognostic risk stratification decision tree to determine adverse events in Anderson-Fabry disease
Source: Eur Heart J Imaging Methods Pract. 2025 May 8;3(1):qyaf032. doi: 10.1093/ehjimp/qyaf032 (PMC12059639; doi:10.1093/ehjimp/qyaf032)
Supplement: qyaf032_Supplementary_Data [file qyaf032_supplementary_data.docx]

**Supplementary Tables**

**Table 1. Demographic data of AFD patients stratified by sex.**

|  | **Males**  **n=22** | **Females n=34** |
| --- | --- | --- |
| **Age (yrs)** | 41.3±14.2 | 40.6±14.2 |
| **Height (cm)** | 175.9±5.8 | 163.9±7.0 |
| **Weight (kg)** | 74.6±14.1 | 69.7±16.4 |
| **BMI (kg/m^2^)** | 24.0±3.8 | 26.0±6.3 |
| **SBP (mmHg)** | 120.0 [18.0] | 110.0 [22.0] |
| **DBP (mmHg)** | 76.5 [10.0] | 73.5 [10.0] |
| **Hypercholesterolemia/Hyperlipidaemia** | 10 (45.5) | 7 (20.6) |
| **Smoker** | 6 (27.3) | 5 (14.7) |
| **Diabetes** | 2 (9.1) | 2 (5.9) |
| **Hypertension** | 9 (40.9) | 4 (11.8) |
| **Chronic kidney disease** | 7 (31.8) | 2 (5.9) |
| **Angina** | 9 (40.9) | 1 (2.9) |
| **Myocardial infarction** | 2 (9.1) | 0 |
| **Heart failure** | 4 (18.2) | 0 |
| **Ventricular tachycardia** | 0 | 1 (2.9) |
| **Atrial fibrillation** | 2 (9.1) | 0 |
| **CVA or TIA** | 2 (9.1) | 1 (2.9) |
| **eGFR (mL/min/1.73m^2^)** | 70.6±27.4 | 85.7±9.4 |
| **ERT treatment at time of scan  (Replagal – agalsidase alfa)** | 3 (13.6) | 1 (2.9) |
| **ERT treatment at time of scan (Fabrazyme – agalsidase beta)** | 2 (9.1) | 1 (2.9) |
| **B-blocker** | 4 (18.2) | 2 (5.9) |
| **ACE/ARB** | 8 (36.4) | 6 (17.6) |
| **Statins** | 6 (27.3) | 6 (17.6) |
| **Diuretic** | 0 | 1 (2.9) |
| **Anticoagulant** | 2 (9.1) | 2 (5.9) |
| **Antiarrhythmic** | 1 (4.5) | 2 (5.9) |

Mean ± standard deviation or median [IQR] for continuous variables; number (percentage) for categorical variables. *BMI, body mass index; BSA, body surface area; CVA, cardiovascular accident; DBP, diastolic blood pressure; SBP, systolic blood pressure; TIA, transient ischaemic attack; yrs, years.*

**Table 2. Receiver operating curve analysis of binary predictors of AFD-related events.**

| **Parameter** | **AUC** |
| --- | --- |
| Increased wall thickness | 0.82 |
| Dilated left atrium (moderate or severe) | 0.74 |
| LV GLS <16% | 0.69 |
| LAS_R_ <24% | 0.66 |

*AUC, area under curve; GLS, global longitudinal strain; LAS_R_, left atrial reservoir strain; LV, left ventricular.*
